# Cost-benefit analysis of Intensive Care Unit with Activity-Based Costing approach in the era COVID-19 pandemic: A case study from Iran

**DOI:** 10.1371/journal.pone.0285792

**Published:** 2023-05-16

**Authors:** Hamed Rahimi, Reza Goudarzi, Nader Markazi-Moghaddam, Amir Nezami-Asl, Sanaz Zargar Balaye Jame

**Affiliations:** 1 Department of Health Management and Economics, Faculty of Medicine, AJA University of Medical Sciences, Tehran, Iran; 2 Health Services Management Research Center, Institute for Futures Studies in Health, Kerman University of Medical Sciences, Kerman, Iran; 3 Faculty of Aerospace and Subaquatic Medicine, AJA University of Medical Sciences, Tehran, Iran; Zayed University, UNITED ARAB EMIRATES

## Abstract

**Background:**

Providing intensive care to acute patients is a vital part of health systems. However, the high cost of Intensive Care Units (ICU) has limited their development, especially in low-income countries. Due to the increasing need for intensive care and limited resources, ICU cost management is important. This study aimed to analyze the cost-benefit of ICU during COVID-19 in Tehran, Iran.

**Methods:**

This cross-sectional study is an economic evaluation of health interventions. The study was conducted in the COVID-19 dedicated ICU, from the provider’s point of view and within one-year horizon. Costs were calculated using a top-down approach and the Activity-Based Costing technique. Benefits were extracted from the hospital’s HIS system. Benefit Cost ratio (BCR) and Net Present Value (NPV) indexes were used for cost-benefit analysis (CBA). A sensitivity analysis was performed to evaluate the dependence of the CBA results on the uncertainties in the cost data. Analysis was performed with Excel and STATA software.

**Results:**

The studied ICU had 43 personnel, 14 active beds, a 77% bed occupancy rate, and 3959 occupied bed days. The total costs were $2,372,125.46 USD, of which 70.3% were direct costs. The highest direct cost was related to human resources. The total net income was $1,213,314.13 USD. NPV and BCR were obtained as $-1,158,811.32 USD and 0.511 respectively.

**Conclusion:**

Despite operating with a relatively high capacity, ICU has had high losses during the COVID-19. Proper management and re-planning in the structure of human resources is recommended due to its importance in the hospital economy, provision of resources based on needs assessment, improvement of drugs management, reduction of insurance deductions in order to reduce costs and improve ICU productivity.

## Introduction

In health systems, hospitals are considered the most important and expensive service providers [1]. Although hospitals account for half of the resources of the health sector [2], the rapid increase in health costs has faced them with continuous and serious financial challenges [3, 4]. In addition, in recent years, the outbreak of COVID-19 has imposed economic burdens and costs on governments, health systems, hospitals, and people [5]. Especially providing services to COVID-19 patients in the Intensive Care Unit (ICU) is very expensive due to the long stay, isolation and complexity of care [6]. So that Ghaffari et al. in their case study in 2019 estimated the economic burden of this disease in Iran for hospitalized patients at 1,439,083,784 dollars [7]. In another study, healthcare costs related to COVID-19 were estimated at 4.26 billion Chinese yuan (US$0.62 billion) in China [8].

Iran is one of the top 5 countries in the world and the first among the Eastern Mediterranean countries in terms of the damage caused by COVID-19 [9]. According to the statistics of the World Health Organization, from January 3, 2020 to February 10, 2023, Iran had 7,565,025 cases of infection and 144,768 deaths due to COVID-19 [10]. Tehran, as the capital of Iran, which constitutes about 20% of Iran’s population [11], has the highest rate of prevalence and death with a growing trend [9, 12]. Evidences have reported different percentages of the hospitalization rate of COVID-19 patients in Iran in ICU; Adham et al. 2.2%, Ghaffari Darab et al. 7% and Shabanpur et al. 17% [7, 13, 14].

In this regard, the ICU, as one of the main departments of providing services to acute COVID-19 patients, is considered one of the most expensive departments of the hospital [4]. So that it accounts for almost 30% (one-third) of hospital expenses [15]. Caring for critical patients in the ICU is associated with a large societal burden. The high cost of treating critical patients in the ICU mainly requires highly trained staff, expensive modern equipment, and intensive use of diagnostic tests, medications, and specific interventions [16]. In addition, the costs caused by COVID-19 for the ICU have been significant [17]. So that the cost of one day of hospitalization of COVID-19 patients in the ICU is estimated to be 3 to 4 times higher than in other departments [7]. Therefore, due to the limited resources and the increasing need for them, the evaluation and management of ICU costs are of great importance [18]. In addition, reliable data about how resources are used and costs related to the COVID-19 disease are very important for health authorities to make decisions [17].

On the other hand, sufficient and sustainable financing is one of the basic needs of strong health systems, especially hospitals [19]. This issue has caused policymakers, managers, and health economists all over the world to search for a way to control and reduce costs [20]. In this regard, it is necessary to evaluate and control the performance of hospitals as the main consumer of health system resources. Especially the financial evaluation that helps hospital managers to determine the current situation, planning to improve performance and financial effectiveness [2]. Because the first and most important step in the process of costs control without losing quality is identifying and knowing the cost of the services we provide [18].

Economics has introduced several approaches and methods for financial evaluation. One of these methods is Cost-Benefit Analysis (CBA). This method compares the expected or achieved benefits of a project with the costs of its implementation [21]. In this context, Activity-Based Costing (ABC) is considered a suitable and widely used approach to accurately identify costs [22]. Therefore, this study was conducted to analyze the cost-benefit of the intensive care unit during the COVID-19 era with an ABC approach, with the aim of knowing the costs and revenues and determining the amount of profit or loss.

## Materials and methods

### Study design and perspectives

The current study is a cross-sectional study of the economic evaluation of health interventions. The research environment is the ICU of a hospital in Tehran, Iran, which was dedicated to patients with COVID-19. The present CBA has been performed on the costs and income extracted from the perspective of the health care provider (hospital) and ABC in the time horizon of one year (from 20 march 2020 to 20 march 2021). The reason for choosing the provider approach in this study is the nature of ICU care and the limited access to data.

According to the theory of microeconomics, stakeholders seek to optimize the ratio of final utility to final cost, and CBA is used to implement this principle for non-saleable goods and services such as health sector programs. CBA is considered to be the most comprehensive economic evaluation method by economists and policymakers because it provides the possibility of the return of investment in health with the return of investment in other economic sectors. Compared to other economic evaluation methods (cost-effectiveness and cost-utility), CBA has the advantage that it can inform policymakers about the monetary value of a program or service and has great potential value in guiding policymakers to allocate and distribute health resources [23]. Also, the ABC method is a cost information system that with covers all stages of activities, including planning, implementation, and support, provides complete information to enable policy makers to process activities and make decisions [24]. Compared to traditional methods, ABC can be evaluated as a suitable basis for performance-based payment, hospital service tariffing, cost control, operational budgeting, and outsourcing and privatization of services in addition to identifying real costs.

### Data collection

Carrying out a CBA includes two parts: cost and benefit. To collect cost data, a checklist, direct observation, and interview with hospital personnel were used. Also, the benefits were extracted from the Health Information System (HIS) of the hospital by referring to the financial management and insurance department. To ensure the accuracy of the data, the data was checked and confirmed by the hospital managers.

### Costs

To calculate costs, the top-down approach and macro costing were used. First, the activity centers were identified and determined through observation and interviews with the hospital officials or the HIS of the hospital. The activity centers are divided into three groups according to operations: final activity center (ICU), intermediate activity centers (paraclinical units including pharmacy, radiology, laboratory, nutrition, and laundry), and overhead activity centers (including financial and accounting units, facilities, management, information technology, and guards). By identifying and tracking operational activities and performing activity analysis, the output type of each activity center was determined, and costing operations were performed based on the outputs.

By defining the above steps, the resources used in each of the outputs, identifying different cost items, and collecting related information, a cost analysis was done in the activity centers. In this stage, 5 groups of costs were identified and calculated: building cost, equipment, and capital goods cost, personnel cost, consumer goods cost, and city facility cost. To allocate indirect costs (intermediate and overhead centers) to the final activity center, the direct allocation method was used. To calculate the depreciation costs of property and equipment, first, a list of equipment prices was prepared and with the opinion of the medical equipment unit experts their installment value and useful life were estimated from reliable sources. For items whose prices were not available, revaluation was used by matching the market-day price. Also, the straight-line method was used to calculate the depreciation cost of capital goods. Considering that the life of the building was more than 20 years, the depreciation cost of the building was considered zero. In this way, the costs of each activity center were determined.

Finally, the cost of the overhead activity centers was distributed to the intermediate and final activity centers. Then the costs of different centers (intermediate and overhead) were allocated to the final cost center (ICU). To share the costs, the basis of proportional sharing of the services of each high-level center was used. For example, accounting and recruitment cost sharing was used based on the number of personnel in each unit, energy and facilities based on infrastructure, management, security, and administration based on the number of employed people, laundry based on kg of washed clothes, and CSR based on the number of sterile packs. In this way, the total cost of the ICU was obtained according to

Totalcost=∑((Directcost=Humanresources+Drugs+Hoteling+Energy+Medical/non‐medicalconsumables+MaintenanceandRepair+Depreciationofbedandequipment)+(Indirectcost=Overheadcosts+Allocatedcostsfromintermediateactivitycenters)) (1)

The costs and incomes were calculated based on the purchasing power parity (PPP) of the dollar and the US dollar, which are 30,000 and 42,000 rials for 1 dollar, respectively, according to the statistics of the World Bank and the Central Bank of Iran [25, 26].

### Benefits

To calculate the benefits, the financial management department of the hospital was consulted and the income of the special care department and the number of insurance deductions were extracted from the HIS system of the hospital. Then, by deducting insurance deductions from the gross income of the department, the net and collected income of the ICU was calculated

Netincome=Totalincome−Insurancedeductions (2)

### Cost-benefit analysis

To check cost-benefit, Benefit Cost ratio (BCR) and Net Present Value (NPV) were calculated using the obtained cost and benefit data

$$BCR=\frac{Present\;Value\;(Benefits)}{Peresnt\;Value\;(Costs)}$$ (3)

$$NPV=\sum_{0}^{n}(\frac{{Benefits}_{\left(t\right)}-{Costs}_{\left(t\right)}}{{\left(1+r\right)}^{t}})$$ (4)

Where t represents the time and r represents the discount rate, which was considered as one and zero, respectively. BCR means how much benefit we have per unit of cost. The condition that resources prevail over costs is that BCR is greater than 1 and NPV is positive [27].

### Sensitivity analysis

Furthermore, to evaluate the dependence of the final results of the CBA on the uncertainties in the cost data, a one-way sensitivity analysis was used. Analysis was performed using Excel-2016 and STATA-12 software.

### Ethical statement

The study design has been approved by the AJA University of Medical Sciences (Grant No: 97000862). Also, this study has been approved by Ethics Committee of the AJA University of Medical Sciences under the ethical code of IR.AJAUMS.REC.1399.253 (webpage of ethical approval code is: https://ethics.research.ac.ir/IndexEn.php). In order to collect data, verbal informed consent was obtained from the senior managers of the hospital. The authors declare that they have no conflict of interests.

## Results

### General characteristics

At the time of the study, the ICU of the studied hospital had 43 personnel, 14 active beds, 77% bed occupancy rate, and 3959 occupied bed days.

### Economic analysis

According to the findings, the total costs of the ICU were $2,372,125.46 USD ($3,320,975.64 PPP). The results showed that 70.3% of ICU costs were related to direct costs ($1,667,604.2 USD, $2,334,645.87 PPP) and 29.7% were related to indirect costs ($704,521.26 USD, $986,329.76 PPP) (Table 1).

**Table 1:** Costs and benefits of ICU In the era of COVID-19, Tehran, Iran.

Items	USD $	PPP $	%	
**Costs**				
**Direct cost**	1,667,604.2	2,334,645.87	100	70.3
Human resources	945,166.27	1,323,232.77	56.68	
Drugs	348,020.85	487,229.2	20.87	
Hoteling	245,894.18	344,251.85	14.75	
Energy (water, electricity, gas)	17,387.76	24,342.86	1.04	
Medical / non-medical consumables	31,209.47	43,693.26	1.87	
Maintenance and Repair	380.95	533.33	0.02	
Depreciation of bed and equipment	79,544.72	111,362.6	4.77	
**Indirect cost**	704,521.26	986,329.76	100	29.7
Overhead costs	514,300.52	720,020.73	73	
Allocated costs from intermediate activity centers	190,220.74	266,309.04	27	
**Total cost**	**2,372,125.46**	**3,320,975.64**	**100**	
Insurance deductions	24,761.51	34,666.12	2	
**Benefits**				
Total income	**1,238,075.65**	**1,733,305.91**	**100**	
Net income	1,213,314.13	1,698,639.8	98	
**Cost-Benefit Analysis**				
Net Present Value (NPV)	-1,158,811.32	-1,622,335.85		
Benefit Cost Ratio (BCR)	0.511			

According to the data in Table 1, human resources costs constitute the largest part of direct costs with 56.68 percent ($945,166.27 USD, $1,323,232.77 PPP). After human resources, drugs cost accounts for the highest percentage of the direct cost of the ICU ($348,020.85 USD, $487,229.2 PPP). Furthermore, the results of the study indicate that 73% of the indirect costs of the ICU of this hospital in 2019 are related to overhead costs ($514,300.52 USD, $720,020.73 PPP) (Table 1).

The results showed that the total gross income of the ICU was $1,238,075.65 USD ($1,733,305.91 PPP). Considering 2% of insurance deductions, the amount of net income of the ICU was $1,213,314.13 USD ($1,698,639.8 PPP). The cost-benefit analysis showed that the BCR index of the ICU is equal to 0.511, and its NPV index is $-1,158,811.32 USD ($1,622,335.85 PPP). In other words, the ICU of this hospital had a loss of $1,158,811.32 at the time of the study (Table 1).

### Sensitivity analysis

**Fig 1:**
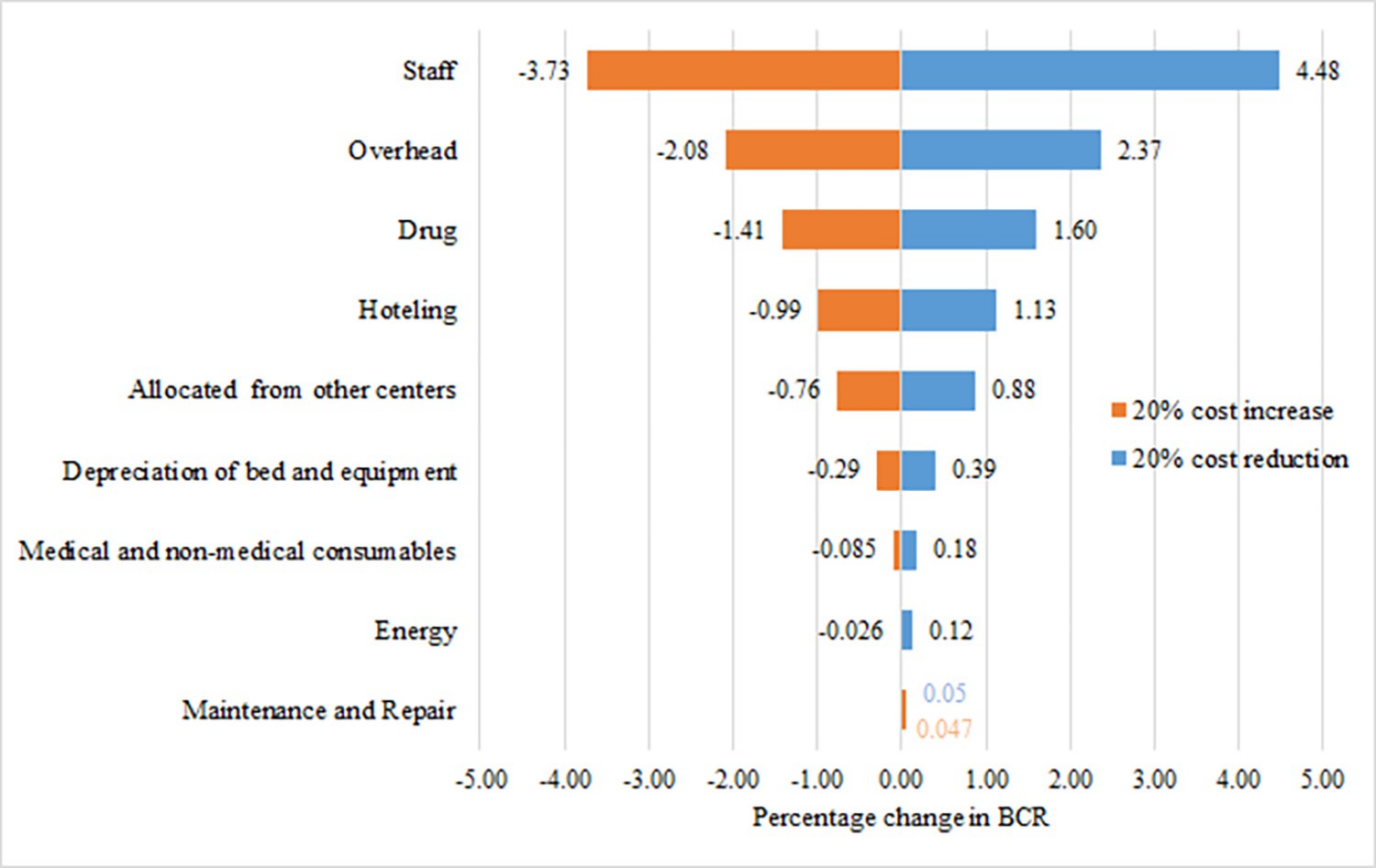
Tornado diagram for the BCR sensitivity analysis.

The results of one-way sensitivity analysis showed that the human resources, overhead, drugs, and hoteling costs were the most sensitive parameters respectively, and caused the most changes in BCR and NPV indices. As Fig 1 shows, with a 20% reduction in human resources costs, the BCR index will increase by 0.0448. Also, with a 20% reduction in overhead costs, the BCR index will grow by 0.0237.

**Fig 2:**
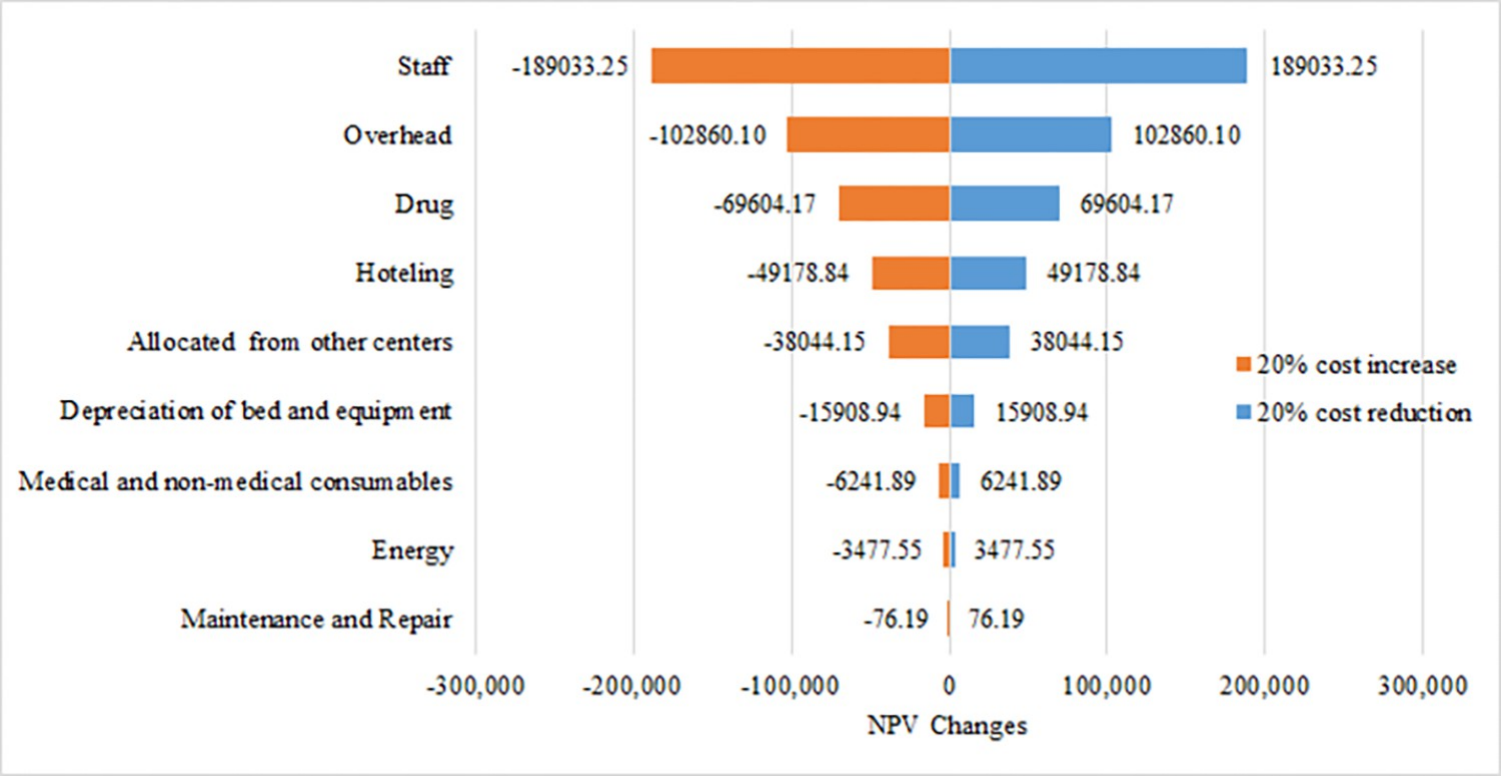
Tornado diagram for the NPV sensitivity analysis.

Furthermore, chart 2 shows that with a 20% reduction in human resources costs, the NPV index will increase by $189,033.25 USD. Also, with a 20% reduction in overhead costs, the NPV index will grow by $102,860.1 USD (Fig 2).

## Discussion

This study was conducted to calculate the total cost and cost-benefit analysis of the ICU in one of Tehran’s hospitals. The total cost was calculated using the direct allocation method based on cost sharing. The total cost of the ICU was estimated to be $2,372,125.46 USD, which included $1,667,604.2 USD direct costs (70.3%) and $704,521.26 USD indirect costs (29.7%). The available evidence confirms these findings, so that in previous studies, from 70 to 97.4 percent of the ICU costs were related to direct costs [20, 28, 29]. In the studied hospital, human resources at 56.68%, drugs at 20.87%, and hoteling at 14.75% accounted for the largest share of direct costs, respectively. Many studies are concordant with the present findings. In most of them, most of the direct costs of ICU were related to human resources wages, which variable from 38% to 71% [20, 28–33]. Furthermore, the results of the study showed that 73% of the indirect costs of ICU were related to overhead costs. Review of the literature shows that the ratio of overhead and intermediate costs is different in different studies. in line with the present study, in Noori et al.’s study (2016), 74% of the indirect costs of the ICU were related to overhead costs [20]. However, in some studies such as Khani et al.’s (2013) and Rezapour et al (2012), intermediate costs constituted 55% and 88% of indirect ICU costs, respectively [29, 30].

In general, the amount of ICU costs and their composition depends on several factors, including the number of beds, occupancy rate, length of stay, admission diagnosis, and the severity of illness [18, 34]. However, the comparison of the findings shows that during the COVID-19 pandemic, the composition and type of ICU costs did not differ from before the pandemic. Especially, the study by Noori et al. was done in this same hospital before the COVID-19 pandemic. Variable costs include factors that are directly related to patient costs (direct cost). On the other hand, ICU overhead costs as major part of indirect costs are almost non-changeable costs and therefore difficult to change [35].

Considering that a significant part of the direct cost of ICU is related to human resources, it is necessary to pay attention to the available human resources to reduce the costs of this department and ultimately the hospital. Factors such as the provision of human resources without basic needs assessment, lack of appropriateness of job position with ability and education, lack of sufficient skills and motivation, lack of proper on-the-job training, lack of opportunities for job rotation and job promotion can affect the reduction of manpower productivity and increase the cost of providing medical services [29, 30]. Cao et al. (2006) point out that although approximately 88% of total ICU costs are related to human resources, equipment, and materials (drugs and medical consumables), reducing the number of staff and equipment is not a good option to maintain and improve the quality of care in the ICU [33]. Among the direct costs, drugs costs are very important, because the growth of ICU drugs costs is higher than that of non-ICU drugs [34]. This issue has significantly affected the field of critical care [36]. Therefore, it will be useful and effective to apply the correct strategy in the supply, storage, and use of pharmaceuticals to prevent and reduce unnecessary costs and apply insurance deductions.

According to the findings, the total cost of ICU was estimated at $2,372,125.46 USD, and on the other hand, the income of this department after deducting insurance deductions was $1,213,314.13 USD. The CBA showed that the ICU was not profitable during the study period. So that this issue was deduced with a value of less than 1 benefit-cost ratio index (0.511) and a negative NPV. This shows that during the outbreak of COVID-19, the monetary value (benefits) of the ICU is much lower than the costs incurred for the provision of medical services to patients. Overall, the ICU has been economically disadvantaged during the COVID-19 era, with costs (taking into account indirect or hidden costs) exceeding revenues. The results of the study by da Silva Etges et al. (2021) in Brazil have shown that the average daily income of the hospital has decreased by 10% during the COVID-19 period. Also, their results indicate that the average investment of the hospital for each hospitalized patient with COVID-19 was 6,800 dollars [37]. The study of Oksuz et al. (2021) in Turkey have shown that the average cost of a COVID-19 patient in the ICU was $14,388.1 (PPP), and the largest share of the ICU costs was related to procedural packages (72%). Their study showed that the direct costs of the disease depend on the severity of the disease and the clinical and demographic characteristics of the patients [38]. Also, the results of Sepúlveda et al.’s (2019) study in Chile showed that ICU suffered a loss of 3,125,393,247 pesos, equivalent to 4,458,822 euros [28]. Also, Cao et al.’s study (2006) in Japan has shown that ICU suffering financial deficit ($383,008) [33].

Evidence suggests that costs decreases with longer ICU stay [18]. For example, Kılıç et al.’s (2019) results showed that for patients with 1, 2, and 3 days of hospitalization in the ICU, the total service costs are more than the hospital’s receipts. In other words, the ICU cost per patient was higher than the patient billing costs. Also, the benefit-loss ratio per patient was more in favor of loss in patients who were hospitalized for 1 day in ICU compared to patients who were hospitalized for 2 and 3 days. However, no loss was observed in patients who stayed in the ICU for 4 or more days. In these patients, the total cost paid by the hospital was higher than the total cost of services per patient. Therefore, the profit/loss ratio was in favor of profit [35]. Meanwhile, a study on COVID-19 patients in the United States showed that with the increase in the stay of these patients in the ICU, hospital costs will also increase. So that their results showed that one day of reduction in the length of stay in the ICU of COVID-19 has an average saving of $3,586 per patient/day. In their study, the median total ICU cost was $13,443 [17].

Although the evidence suggests that a few studies have been conducted on costs and cost-benefit analysis of ICUs, especially in low- and middle-income countries [32], but shows that ICU is expensive. So, the daily cost of an ICU bed is three times that of a general bed [4, 18]. This unit has the highest cost of equipment depreciation, the highest cost of consumables and special materials, the highest variable costs, the highest cost per service unit, the lowest amount of income per service unit, the highest operating loss per service unit, and the pharmaceutical cost is higher than to other treatment departments of the hospital [30, 31, 34]. One of the main reasons for the high cost of ICU is the follow-up of patients with complex and serious diseases, the number of personnel, the use of expensive tools and equipment, and the high consumption of drugs, laboratory tests, and imaging [35].

Considering the 77% ICU bed occupancy rate, it can be said that its capacity is not fully used. Since the increase in the bed occupancy rate leads to a decrease in hospital costs [39], the management’s effort in using appropriate strategies to improve the ICU bed occupancy rate can reduce its costs to some extent and improve its productivity. Also, one of the strategies facing hospital managers to reduce costs can be remote ICU. The findings of the study by Michael Robie et al. (2022) showed that remote ICU helps to reduce hospital service costs by 36% and positive financial results of ROI and cost-effectiveness. The implementation of the Tele-ICU program has had a positive impact on financial and clinical areas, reducing mortality and hospital length of stay. Thus, the reduction of length of stay through remote ICU has saved up to 11.5 million dollars [40]. Additionally, it has improved patient safety, and quality of care, and reduced ventilator days, making it a cost-effective technology [41]. Therefore, by having a clear vision and proper planning, health managers can implement this technology and benefit from its benefits.

Also, one of the solutions that the world economy has achieved to avoid costs is outsourcing [42]. Because outsourcing helps shift fixed costs to variable costs [43]. While this strategy has been successful in reducing costs of many organizations, but many believe that applying this strategy to clinical services is associated with risks [44]. So that some evidence of the conservativeness of hospitals in outsourcing their clinical services has been reported [45, 46]. Young also believes that outsourcing clinical services not only does not save money but also creates many risks for patients [46]. Mehdizadeh et al. (2016) also believe that the weaknesses of hospitals in outsourcing clinical services are more than their strengths. It is also very important to pay attention to the health of patients and their rights when outsourcing clinical services [47]. Therefore, the outsourcing decision is complex and needs to evaluate its various aspects logically [48–50], and outsourcing cannot be done solely for economic reasons and to reduce costs.

The most important limitation of this study was the COVID-19 pandemic, the imposition of quarantine, and traffic restrictions, which led to the disruption of the data collection process. Another limitation was recording and reporting cost information jointly for the entire hospital. Therefore, in this study, cost-sharing methods were used to allocate these costs to the ICU, where the calculated costs may deviate from the actual amount. Also, the lack of access to the price of some equipment was one of the limitations of this study. Therefore, the price of this equipment was obtained through an interview with the medical equipment manager of the hospital and the web pages of the medical equipment companies. Furthermore, in this study, the cost of equipping and setting up the ICU was not considered, and the study was limited to the cost-benefit analysis of one year of ICU activity.

## Conclusion

Based on the results of the study, despite operating with relatively high capacity, the ICU has had a high loss during the COVID-19 era. Proper management and re-planning in the structure of human resources is recommended due to its importance in the hospital economy, provision of resources based on needs assessment, improvement of drugs management, and reduction of insurance deductions to reduce costs and improve ICU productivity.

## Supporting information

S1 Appendix (DOCX)

S2 Appendix (XLSX)
