# Supplementary material for: Cost-benefit analysis of Intensive Care Unit with Activity-Based Costing approach in the era COVID-19 pandemic: A case study from Iran
Source: PLoS One. 2023 May 16;18(5):e0285792. doi: 10.1371/journal.pone.0285792 (PMC10187905; doi:10.1371/journal.pone.0285792)
Supplement: S1 Appendix — (DOCX) [file pone.0285792.s001.docx]

**Data collection form**

**Cost-Benefit Analysis of Intensive care unit (ICU)**

**Direct costs of the ICU**

| **Final Center** | Human resources | Drug | Hoteling | Energy | Medical / non-medical consumables | Maintenance and Repair | Depreciation of bed and equipment |
| --- | --- | --- | --- | --- | --- | --- | --- |
| **ICU** |  |  |  |  |  |  |  |

**Indirect costs:**

| **Center type** | **Name of the centers** | **Basis of cost sharing** | **cost** | **Cost allocated to ICU** |
| --- | --- | --- | --- | --- |
| **Intermediate activity centers** | Operating room | Number of admissions |  |  |
|  | Emergency | Number of admissions |  |  |
|  | Pharmacy | Number of admissions |  |  |
|  | Medical imaging | Number of admissions |  |  |
|  | Laboratory | Number of admissions |  |  |
|  | Total | |  |  |
| **Overhead activity centers** | Laundry | Washed clothes (kg) |  |  |
|  | Nutrition | Bed day |  |  |
|  | Energy | Infrastructure (space) |  |  |
|  | Management & administrative | Number of employed people |  |  |
|  | Public Service | Infrastructure (space) |  |  |
|  | guarding | Number of employed people |  |  |
|  | Central Sterilization Room (CSR) | Number of sterile packs |  |  |
|  | Facility engineering | Infrastructure (space) |  |  |
|  | Total | |  |  |

Total cost: ……………………………….

Income of the ICU department: ……………………...

Insurance deductions: ……………………
